# Motor rehabilitation should be based on knowledge of motor control

**DOI:** 10.1186/s40945-016-0019-z

**Published:** 2016-06-23

**Authors:** Daniele Piscitelli

**Affiliations:** School of Medicine and Surgery, PhD Program in Neuroscience, University of Milan-Bicocca, Milan, Italy

**Keywords:** Motor control, Stability, Synergy, Movement disorders, Neurorehabilitation

## Abstract

Neurorehabilitation is at a crossroads. Indeed, there is inconclusive, but promising evidence about clinical effectiveness of rehabilitation in the field of neurological impairments. Translating the new theories on motor control into clinical research may help to develop new treatment strategies and guide rehabilitation approaches. The concepts of synergy and the uncontrolled manifold hypothesis provide a strong theoretical framework to explain how the nervous system controls and coordinates movements, ensuring stability during daily actions. Moreover, this approach can increase the understanding of the neural control of action stability with implications for clinical practice and may help the development of new treatment strategies.

## Main text

Motor control is an area of natural science exploring how the nervous system interacts with other body parts and the environment to produce purposeful, coordinated actions. It is a growing scientific field with strong implications for neurological movement disorders and development of treatment strategies.

One of the central problems in the field of neurorehabilitation is uncertainty about the best type of rehabilitation or physiotherapy interventions among patients with neurological impairments. There have been only a few definitive multi-site randomized clinical trials published over the last years [1–6]. Most of these trials [1, 3–6] reported disappointing results, i.e. no differences in the improvement in the primary outcome variables between the experimental and control group. Moreover, a recent clinic trial [7] investigated the effectiveness of physiotherapy and occupational therapy in mild to moderate Parkinson disease revealed that the group receiving therapy did not show immediate or medium-term improvements in activities of daily living or quality of life. These poor outcomes could be due to the lack of translating the current theories of the neural control of movement into clinical research settings. The underlying mechanisms and the changes of motor control should be considered as ingredient to guide and develop rehabilitation approaches.

An important aspect of everyday movements is their stability. A movement lacking stability is functionally useless given the changing and unpredictable intrinsic body states and external forces.

Recently, a hypothesis has been proposed that the central nervous system is able to organize elements within the body into task-specific ensembles (synergies) stabilizing salient performance variables: the “uncontrolled manifold” (UCM) hypothesis [8]. This hypothesis assumes that processes associated with voluntary movements can be adequately expressed as creating a sub-space (UCM) in the space of elemental variables corresponding to a desired value of an important performance variable to which all the elements contribute. For example, during multi-digit prehension, individual digit forces and moments (i.e. elemental variables) co-vary to stabilize the resultant force/moment (i.e. performance variable) acting on the grasped object. Therefore, given a particular value of a task variable (e.g. pointing, reaching), the UCM is the set of all joint configurations that lead to that same value of the task variable.

The concepts of synergy and UCM are intimately linked to a feature of the human body addressed as redundancy [9]: at any level of description of human movements, there are more elements (such as muscles, joints, etc.) than minimally necessary to perform typical tasks. For example, one can place the index fingertip into a target in the three-dimensional space with an infinite number of joint configurations.

Recently, the problem of redundancy has been reassessed based on the principle of abundance [10, 11], which suggests that the apparently redundant design is not a source of computational problems but an essential feature that ensures stability of actions in combination with the possibility of performing actions with the same set of elements.

The UCM hypothesis allows introducing a quantitative and objective measure, synergy index, reflecting how flexible solutions are used to stabilize a particular performance variable. This index reflects the relative amount of inter-trial variance within a subspace (UCM) where this performance variable is unchanged compared to the amount of variance orthogonal to the UCM, where the performance variable changes. It is quantified from the inter-trial variance of elemental variables (e.g. digit forces/moments for prehension) compared the variance that not affect the magnitude of performance value (V_ucm_) and variance that did (V_ort_) [12].

This index has been quantified for multi-digit pressing and prehension tasks, multi-joint reaching tasks, and multi-muscle whole-body tasks [12, 13].

A series of studies have shown an ability of the nervous system to adjust stability properties in preparation for a quick action, leading to an attenuation of the synergy index stabilizing a variable (e.g., change in a force production during an isometric task or generation of postural corrections) in preparation to a quick change in that variable. These feed-forward adjustments have been named anticipatory synergy adjustments (ASAs).

This approach has allowed quantifying impairments in the neural control of action stability across populations of neurological patients including those with Parkinson’s disease (PD), multi-system atrophy, multiple sclerosis, and stroke [14]. In recent studies on patients with early-stage PD, the synergy index and ASAs were both reduced compared to healthy matched subjects. The low synergy index reflects the low stability of steady-state actions in PD, while the inability to attenuate the synergy index prior to an action could be causally related to the well-known clinical signs such as “freezing of gait” in PD. These findings are consistent in other patient populations although mild cortical stroke leads to significantly delayed ASAs without a difference in the synergy index magnitude [15].

So far, studies of the effects of exercise on motor synergies have been limited to a handful of populations including persons with atypical development (i.e. Down syndrome) and healthy elderly (reviewed in Latash & Huang [14]). These studies employed both non-specific training (such as strength training) and exercise designed to challenge action stability and encourage stronger task-specific synergies. The latter studies led to particularly striking effects: A significant increase in the synergy index after 30 min of exercise, which was retained at least for two weeks [16, 17]. Overall, these results suggest that synergies can be improved in populations with impaired control of action stability, in particular in patients with neurological disorders. Unfortunately, to date there have been no studies estimating the changes in motor synergies following specific motor training or traditional rehabilitation interventions.

Taken together, these results are promising to increase our knowledge of some of the salient features of the neural control of movement with possible implications for development of treatment strategies. Future researches should explore how rehabilitation programs may benefit from this theoretical framework and how physical therapy may restore impaired synergies that lead to poor control of movement stability.

## Abbreviations

ASAs, anticipatory synergy adjustments; PD, Parkinson’s disease; UCM, uncontrolled manifold
